# Expression of SARS-CoV-2-related receptors in cells of the neurovascular unit: implications for HIV-1 infection

**DOI:** 10.1186/s12974-021-02210-2

**Published:** 2021-07-29

**Authors:** Silvia Torices, Rosalba Cabrera, Michael Stangis, Oandy Naranjo, Nikolai Fattakhov, Timea Teglas, Daniel Adesse, Michal Toborek

**Affiliations:** 1 Department of Biochemistry and Molecular Biology, University of Miami Miller School of Medicine, 528E Gautier Bldg. 1011 NW 15th Street, Miami, FL 33136 USA; 2 Laboratory of Structural Biology, Instituto Oswaldo Cruz, Fiocruz, CEP, Rio de Janeiro, RJ 21045-900 Brazil

**Keywords:** SARS-CoV-2, Blood-brain barrier, HIV-1, ACE2, TMPRSS2

## Abstract

**Background:**

Neurological complications are common in patients affected by COVID-19 due to the ability of SARS-CoV-2 to infect brains. While the mechanisms of this process are not fully understood, it has been proposed that SARS-CoV-2 can infect the cells of the neurovascular unit (NVU), which form the blood-brain barrier (BBB). The aim of the current study was to analyze the expression pattern of the main SARS-CoV-2 receptors in naïve and HIV-1-infected cells of the NVU in order to elucidate a possible pathway of the virus entry into the brain and a potential modulatory impact of HIV-1 in this process.

**Methods:**

The gene and protein expression profile of ACE2, TMPRSS2, ADAM17, BSG, DPP4, AGTR2, ANPEP, cathepsin B, and cathepsin L was assessed by qPCR, immunoblotting, and immunostaining, respectively. In addition, we investigated if brain endothelial cells can be affected by the exposure to the S1 subunit of the S protein, the domain responsible for the direct binding of SARS-CoV-2 to the ACE2 receptors.

**Results:**

The receptors involved in SARS-CoV-2 infection are co-expressed in the cells of the NVU, especially in astrocytes and microglial cells. These receptors are functionally active as exposure of endothelial cells to the SARS CoV-2 S1 protein subunit altered the expression pattern of tight junction proteins, such as claudin-5 and ZO-1. Additionally, HIV-1 infection upregulated ACE2 and TMPRSS2 expression in brain astrocytes and microglia cells.

**Conclusions:**

These findings provide key insight into SARS-CoV-2 recognition by cells of the NVU and may help to develop possible treatment of CNS complications of COVID-19.

**Supplementary Information:**

The online version contains supplementary material available at 10.1186/s12974-021-02210-2.

## Introduction

Coronavirus disease-19 (COVID-19), which is caused by severe acute respiratory syndrome coronavirus 2 (SARS-CoV-2) was first reported in Wuhan, China, in December 2019. COVID-19 has become a pandemic, resulting in devastating morbidity and mortality worldwide due to lethal pneumonia and respiratory distress [1]. In addition to systemic, respiratory, and cardiovascular complications [2], it has been reported that patients with COVID-19 can manifest symptoms of a central nervous system (CNS) infection, such as impaired consciousness, headache, encephalopathy, delirium, paresthesia, ataxia, encephalitis, as well as acute cerebrovascular events, such as ischemic or hemorrhagic stroke [3–12]. COVID-19 patients also experience rhabdomyolysis [13], myositis [14], Guillain-Barré syndrome (GBS) [15], and taste or olfactory impairment [16, 17]. The incidence of neurological complications is estimated to be around 37% in SARS-CoV-2-infected patients [5].

The SARS-CoV-2 genomic sequence shows a similarity of 75.5% with SARS-CoV-1 [18]. Similar to SARS-CoV-1, SARS-CoV-2 binds to the host cell through the transmembrane protein angiotensin I converting enzyme 2 (ACE2) receptor using the spike protein (S protein). This protein is encoded by the S gene and is formed by the subunits S1 and S2. SARS-CoV-2 attaches to the host cells through the S1 subunit to ACE2 [19]. Once attached, the host infection requires the expression of transmembrane serine protease 2 (TMPRSS2) for priming of the spike protein and viral entry into the cell [20–22]. Along with ACE2 and TMPRSS2, several other molecules have been suggested to participate in SARS-CoV-2 entry into human cells, such as ADAM metallopeptidase domain 17 (ADAM17) [23, 24], dipeptidyl peptidase 4 (DPP4) [25, 26], angiotensin II receptor type 2 (AGTR2) [27, 28], basigin (BSG, also called extracellular matrix metalloproteinase inducer [EMMPRIN] or cluster of differentiation 147 [CD147]) [19, 29], aminopeptidase N (ANPEP), [30], and cathepsin B/L [31, 32].

The central nervous system (CNS) is well documented to be a target of beta coronaviruses infections such as SARS-CoV-1 [33–36] and SARS-CoV-2. Several studies detected SARS-CoV-2 in the brain and the cerebrospinal fluid of COVID-19 patients [5, 37–39]. Some reports sustain that this neuroinvasion is due to retrograde axonal transport of the virus via the olfactory sensory neurons [3, 40, 41]. In support of this notion, expression of ACE2 was demonstrated in human olfactory epithelium [17, 42], and olfactory dysfunction is a common symptom in SARS-CoV-2-infected individuals [5, 43, 44]. Moreover, it was suggested that SARS-CoV-2 may reach the cerebral vasculature through the systemic circulatory system [25, 41] and crossing the blood-brain barrier (BBB). While the mechanisms of this process are not fully understood, it has been suggested that it may be executed by infection of cells that compose the microvessels forming the BBB [45]. The BBB, which is mainly formed by endothelial cells (EC), represents a barrier interface between the systemic circulation and the CNS. Surrounding the microvessels and coordinating function with EC are pericytes, astrocytes, neurons, and microglia forming functional elements of the BBB called the neurovascular units (NVU) [46]. The barrier properties of the BBB are generated by high-resistance interendothelial tight junctions (TJs) formed by transmembrane proteins, such as claudin-5, and cytoplasmic proteins, such as zonula occludens (ZO-1), that limit the paracellular permeability between endothelial cells [47].

Older individuals and people with preexisting medical conditions are at higher risk of COVID-19 complications. These factors may be of particular significance in HIV-1 infection as HIV-1-infected patients experience accelerated aging and may suffer from immunodeficiency. In addition, HIV-1 is known to enter the CNS by altering the structures and properties of the BBB [48]. In the brain, perivascular macrophages and microglia represent the primary cells infected with HIV [49, 50]. Several investigations have also shown the capacity of astrocytes to be infected by HIV-1 [51–53] and recent evidence has emerged on the productive HIV-1 infection of brain pericytes [54–58]. In contrast, no productive HIV-1 infection in EC or neurons has been reported [59, 60].

In the present study, we aimed to analyze the expression profile of the main SARS-CoV-2 receptors in host cells forming the NVU in order to elucidate a possible pathway of the virus entry into the brain. Identifying the NVU cells with the greatest potential to be directly infected by SARS-CoV-2 would allow us to better understand the mechanisms of neuroinvasion and viral pathogenesis of SARS-CoV-2 in the brain. Taking into consideration possible interactions between SARS-CoV-2 and HIV-1, we also evaluated the expression of ACE2 and TMPRSS2, i.e., the main SARS-CoV-2 receptors, in these cells after HIV-1 infection. The obtained results indicate that the receptors involved in SARS-CoV-2 infection are coexpressed in the cells of the NVU, especially in astrocytes and microglial cells. Exposure of endothelial cells to the SARS CoV-2 S1 protein subunit altered the expression of TJ proteins, such as claudin-5 and ZO-1, potentially providing a route of SARS-CoV-2 entry into the brain. Additionally, HIV-1 infection upregulated ACE2 and TMPRSS2 expression in astrocytes and microglial cells. Overall, these findings provide key insight into the SARS-CoV-2 recognition by cells of the NVU and may help to develop possible treatment of CNS complications of COVID-19 disease.

## Material and methods

### Cell cultures

Primary human brain microvascular endothelial cells were obtained from Cell Systems (Kirkland, WA, USA, Cat #ACBRI 376) and cultured in a medium supplemented with CultureBoost, 10% serum, 100 units/mL penicillin, and 100 μg/mL streptomycin. For TJ protein measurements, cells were exposed to 15 nM of the SARS-CoV-2 S protein S1 subunit (RayBiotech, Peachtree Corners, GA, Cat # 230-01101) in serum-free media without added antibiotics. Primary human astrocytes (ScienCell, Carlsbad, CA, USA, Cat #1800) were cultured in an astrocyte-specific growth medium (ScienCell, #1801) supplemented with 2% FBS, astrocyte growth supplement, 100 units/mL penicillin, and 100 μg/mL streptomycin. Primary human brain vascular pericytes (ScienCell, Cat# 1200) were maintained in a pericyte-specific growth medium (ScienCell, Cat# 1201) supplemented with 2% FBS, pericyte growth supplement, 100 units/mL penicillin, and 100 μg/mL streptomycin. Immortalized human microglia cell line (hμglia C20) created by SV40/hTERT-mediated immortalization [61] was kindly provided by Dr. Jonathan Karn (Case Western Reserve University, Ohio, OH, USA). Hμglia C20 cells were cultured as earlier described [62] in BrainPhys medium (StemCell Technologies, Vancouver, BC, Canada, Cat# 05791) containing 1X N2 supplement-A (Thermo Fisher Scientific, Cat #17502–048), 1× penicillin streptomycin (Gibco, Cat #15140122), 100 μg/mL normocin (InvivoGen, San Diego, CA, USA, Cat #ant-nr-1), 25 mM L-Glutamine (Thermo Fisher Scientific, Cat#25030081), 1% FBS, and 1 μM dexamethasone (Sigma-Aldrich, St. Louis, MO, USA Cat #D4902). Human neuroblastoma SH-SY5Y cell line (ATCC, Cat#CRL-2266) and human embryonic kidney (43)-293T cells (ATCC, Manassas, VA, USA, Cat# CRL-11268) were cultured in Dulbecco’s Modified Eagle Medium (DMEM) (Thermo Fisher Scientific, Carlsbad, CA, USA, Cat#11995-065) and supplemented with 10% FBS (ScienCell, Cat# 0500), 100 units/mL penicillin, and 100 μg/mL streptomycin (Thermo Fisher Scientific, Cat# 15140-122). All cell cultures were maintained in 5% CO2 at 37 °C.

### HIV-1 production and infection

The HIV-1 pNL4-3 plasmid was acquired from the NIH AIDS Reagent Program (Division of AIDS, NIAID, National Institutes of Health). Viral stocks were generated by transfecting 10^7^ HEK-293T cells with 30 μg HIV-1 pNL4-3 plasmid using Lipofectamine 2000 (Thermo Fisher Scientific, Cat# 11668-027). The next day, the medium was shifted to Opti-Mem (Thermo Fisher Scientific, Cat# 11058-021). After 48 h, supernatants were collected, filtered using 0.45-μm pore size filter (Millipore Sigma, Massachusetts, MA, USA, Cat# 430314), and concentrated using weight exclusion columns (Millipore Sigma, Cat# UFC905024). Viral stocks were stored at – 80 °C. Primary human brain pericytes, astrocytes, and immortalized human microglial cells were infected by incubation with 60 ng p24 HIV-1/mL for 12 h, followed by extensive washing with PBS to remove the unbound virus before the addition of fresh medium. p24 antigen concentration was measured by using HIV-1 p24 Antigen ELISA 2.0 (Zeptometrix, Buffalo, NY, USA Cat# 0801008) according to the manufacturer’s instructions. HIV-1 infection did not affect the viability of the infected cells (Supplemental Figure 1).

### Cell viability

Trypan blue exclusion assay and Cell Counting Kit 8 (CCK-8) assay (Abcam, Cat# ab228554) were used to determine the effect of HIV-1 infection on the cell viability. For the trypan blue exclusion assay, cells in suspension were mixed 1:1 with a 0.4% solution of Trypan Blue Stain (Invitrogen, Cat#T10282), and 10 μL samples were added to a chamber slide (Invitrogen, Cat# C10228) and inserted into a Countess II FL Automated Cell Counter (Invitrogen). Cell viability was calculated using the ratio of living cells to the total number of cells and expressed as a percentage. The CCK-8 assay was performed as suggested by the manufacturer’s protocol. After incubation of cells with 60 ng/mL of HIV-1 p24 for 24 h or 48 h in a 96-well plate, 10 μL of the CCK-8 solution containing WST-8 was added directly to the cell cultures. The plates were incubated for 2 h at 37 °C in a humidified 5% CO_2_ environment. The WST-8 formazan product proportional to the number of living cells was measured at 460 nm using a SpectraMax 190 Microplate Reader (Molecular Devices LLC).

### Quantitative PCR

Total RNA was isolated from cell culture lysates using RNeasy mini kit (Qiagen, Germantown, MD, Cat # 74104) following the manufacturer’s instructions and quantified using the Nanodrop 2000 (Thermo Fisher Scientific). RT-PCR was performed with a total of 100-800 ng of RNA using the qScript XLT 1-Step RT-qPCR ToughMix Low ROX (Quantabio, Beverly, MA, USA, Cat #89236-676) reaction mix and the Applied Biosystems 7500 system (Applied Biosystems, Foster City, CA). TaqMan Gene Expression Assays and ACE2 primer: Hs01085333_m1; TMPRSS2 primer: Hs00237175_m1; ADAM17 primer: Hs01041915_m1; BSG primer: Hs00936295_m1; DPP4 primer: Hs00897386_m1; AGTR2 primer: Hs02621316_s1; ANPEP primer: Hs00174265_m1; CATHEPSIN L primer: Hs02803063_cn and CATHEPSIN B primer: Hs02148115_cn were used for gene amplification. HIV-1 gag was measured using the following primers and probe: HIV-1gag_F 5′-GACATAAGACAGGGACCAAAGG-3′; HIV-1gag_R 5′-CTGGGTTTGCATTTTGGACC-3′; HIV-1gag_Probe 5′-AACTCTAAGAGCCGAGCAAGCTTCAC-3′. Human GAPDH was calculated for sample normalization. Coveted PCR product specificity was determined using melting curve assessment and gene expression fluctuations were determined by the ΔΔCt method, with Ct as the cycle number at threshold.

### Immunoblotting

After washing with phosphate-buffered saline (PBS), cells were lysed with Radio Immuno Precipitation Assay (RIPA) buffer containing protease inhibitors (Santa Cruz Biotechnology, Dallas, TX, USA, Cat# sc-24948a). Protein concentration was assessed using BCA Protein Assay Kit (Thermo Fisher Scientific, Cat# 23223). Samples were loaded on sodium dodecyl sulfate (SDS) polyacrylamide 4-20% Mini-PROTEAN TGX Stain-Free Protein Gels (Bio-Rad Laboratories, Hercules, CA, USA Cat# 4568094) and electrotransferred to a nitrocellulose membrane using the Trans-Blot Turbo Transfer System (Bio-Rad Laboratories, Cat# 170-4159). Afterward, the membranes were blocked with 5% bovine serum album (BSA) in TBS (Sigma-Aldrich, Cat# A7906-500G) for 1h. The blots were incubated in 5% BSA in TBS, overnight at 4 °C with the following primary antibodies: mouse anti-ACE2 (Abcam, United Kingdom, Cat# ab15348), mouse anti-ZO-1 (Thermo Fisher Scientific, Cat# 339100), rabbit anti-claudin-5 (Thermo Fisher Scientific, Cat#341600) and rabbit anti-TMPRSS2 (Thermo Fisher Scientific, Cat# PA5-14264). Membranes were imaged using the Licor CLX imaging system after incubation with the secondary antibody in 5% BSA-TBS for 1h at room temperature (1:20000, LI-COR, Lincoln, NE, USA, Cat# 926-32210, Cat# 926-68070, Cat# 926-32211, Cat# 926-68071). Target protein levels were normalized to anti-GAPDH (1:20000, Novus Biologicals Cat# NB600–502FR or Cat# NB600-5021R) or rabbit polyclonal anti-α-tubulin antibody (1:1000, Thermo Fisher Scientific, Cat# 11224-1-AP). Signal quantification was performed using Image Studio 4.0 software (LI-COR).

### Immunostaining

Cells were seeded on glass coverslips coated with Poly-L-Lysine (Sigma, Cat# P8920-100ML) at a density of 10^5^ cells per cm^2^ after. Cells were then fixed in a 4% paraformaldehyde solution for 15 min, permeabilized using 0.1% Triton-X in PBS for 15 min, and blocked with 3% BSA in TBS for 1 h. Samples were then incubated overnight at 4 °C with 1:100 anti-ACE2 antibody (Abcam, Cat# ab15348) or 1:50 anti-TMPRSS2 antibody (Santa Cruz Biotechnology, Cat# sc-515727) diluted in 3% BSA in TBS. The next day, cells were washed three times with PBS and the samples were incubated with 1:300 in 3% BSA in TBS Alexa Fluor 488- secondary antibody (Thermo Fisher Scientific, Cat# A11034) for 1 h. Cells were washed five times with PBS and then mounted on glass slides using Vectashield Antifade Mounting Medium with DAPI to visualize the nuclei (Vector Laboratories, Burlingame, CA, USA, Cat# H-1500). Images were taken using the Olympus FluoView 1200 Laser Scanning Confocal Microscope (Olympus, Center Valley, PA, USA).

### Statistical analysis

All statistical analyses between experimental groups and controls were performed with GraphPad Prism 6 (GraphPad Software, La Jolla, CA, USA). Two-tailed Student’s *t*-test or one-way ANOVA following Turkey’s multiple comparisons test were used for the analysis and *p* < 0.05 was considered significant.

## Results

### Cells of the NVU differentially express receptors involved in SARS-CoV-2 infection

**Fig. 1:**
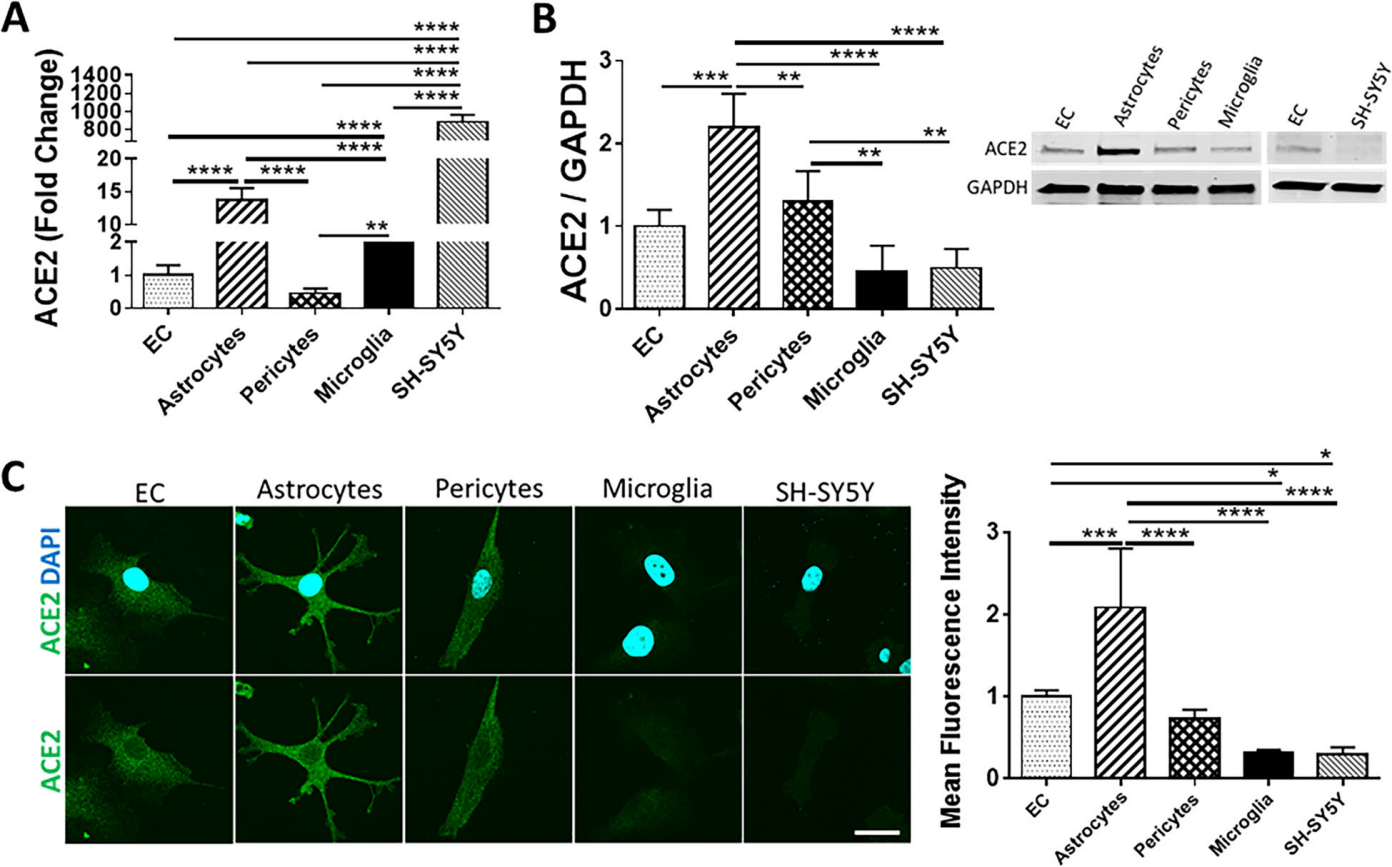
Expression of ACE2 in cells of the NVU. Expression levels of ACE2 were measured by q-PCR (**A**), immunoblotting (**B**), and immunostaining (**C**). GAPDH was used as a housekeeping gene and loading control. Graphs indicate the mean ± SD from three independent experiments. ****p < 0.0001, ***p = 0.0002, **p = 0.003, *p < 0.0449, *n* = 3–6 per group; scale bars, 30 μm

We first sought to characterize the expression levels of the main molecules involved in SARS-CoV-2 entry into host cells. RNA and protein were extracted from primary human brain EC, astrocytes, pericytes, immortalized human microglial cells, and SH-SY5Y neuroblastoma cell line. Expression of ACE2, TMPRSS2, ADAM17, BSG, DPP4, AGTR2, ANPEP, cathepsin L, and cathepsin B were analyzed by qPCR, immunoblotting, and immunostaining. ACE2 was significantly more expressed at mRNA levels in SH-SY5Y cells compared to other cells of the NVU. Astrocytes were characterized by the second higher expression of ACE2 at mRNA levels but the highest protein expression of this receptor. Microglial cells presented a modest expression of ACE2 both at mRNA levels and low at protein levels. EC and pericytes exhibited the lowest mRNA expression of ACE2 but higher protein levels than microglial cells (Fig. 1 A and B). The differences in protein expression detected by immunoblotting were fully confirmed by immunostaining (Fig. 1C).

**Fig. 2:**
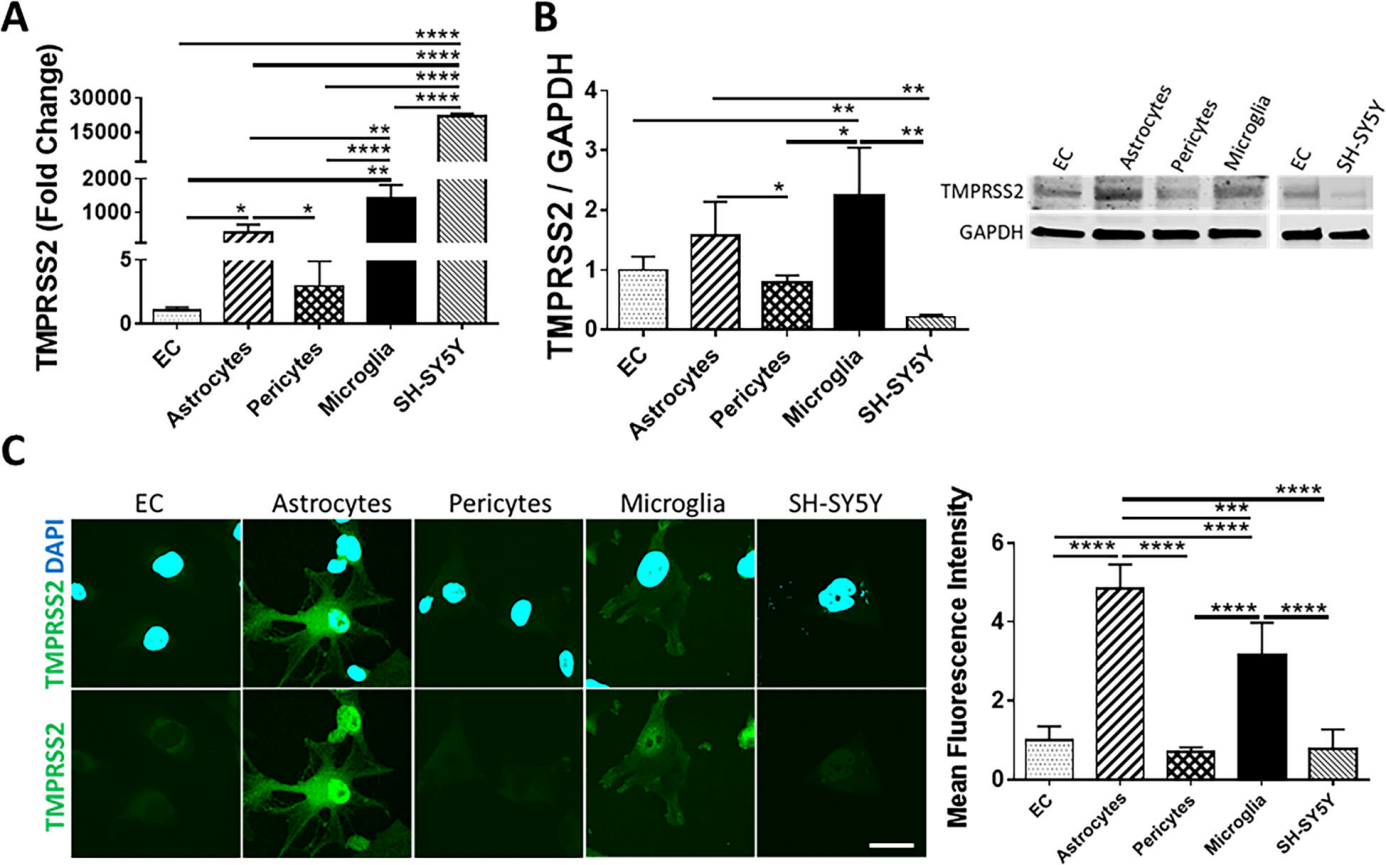
Expression of TMPRSS2 in cells of the NVU. Expression levels of TMPRSS2 were measured by q-PCR (**A**), immunoblotting (**B**), and immunostaining (**C**). GAPDH was used as a housekeeping gene and loading control. Graphs indicate the mean ± SD from three independent experiments. ****p < 0.0001, ***p = 0.0002, **p = 0.003, *p < 0.0449, *n* = 3–6 per group; scale bars, 30 μm

Among studied cells, SH-SY5Y cells expressed the highest TMPRSS2 mRNA levels but the lowest protein levels. Microglial cells and astrocytes demonstrated the second highest mRNA levels and the highest TMPRSS2 protein expression (Fig. 2A–C). The lowest expression of TMPRSS2 mRNA levels were detected in EC, followed by pericytes (Fig. 2A). These cell types also expressed low levels of TMPRSS2 at the protein levels (Fig. 2 B and C). These results were confirmed by immunostaining (Fig. 2C).

**Fig 3:**
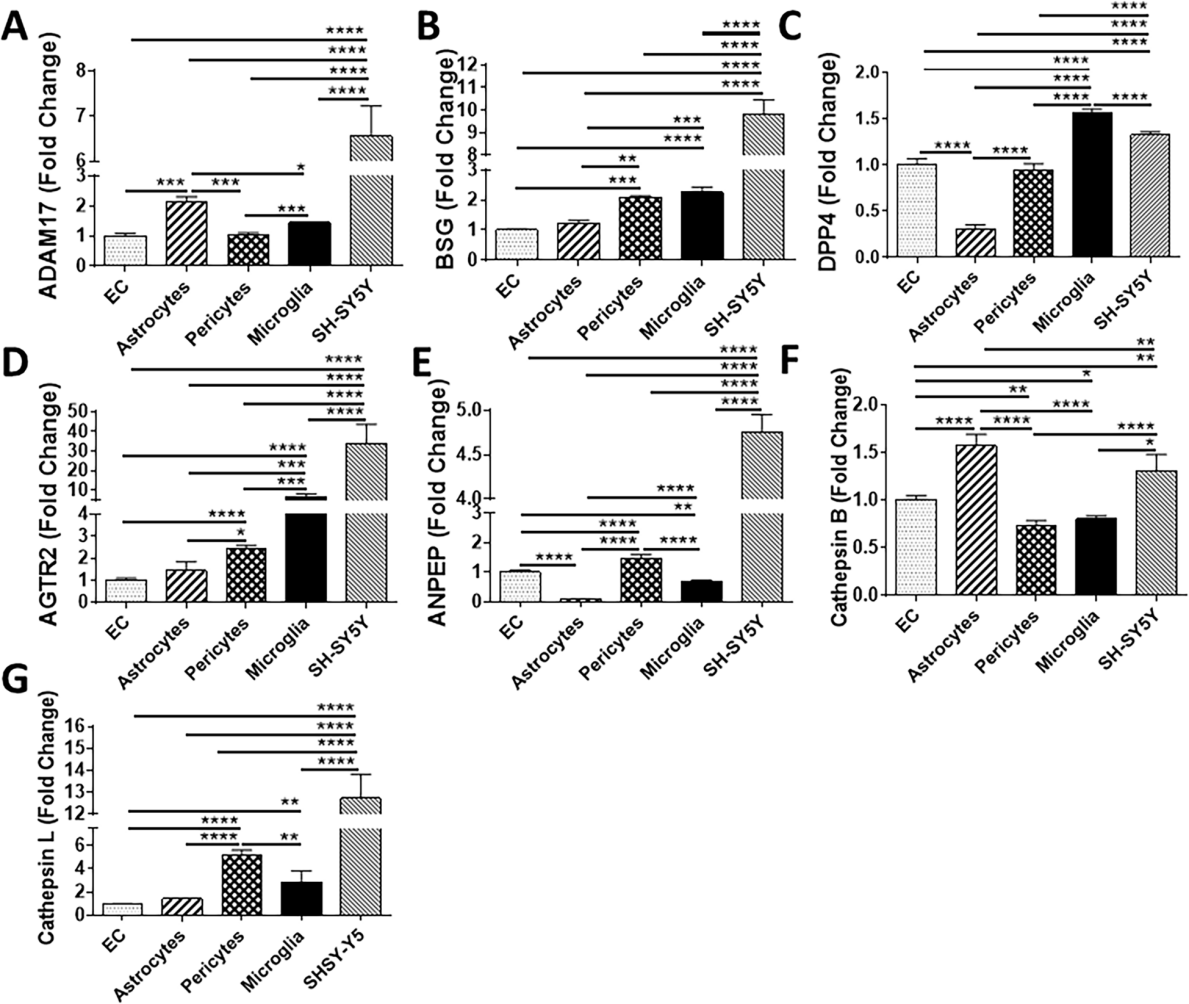
Expression of SARS-CoV-2 entry molecules in cells of the NVU. Expression levels of ADAM17 (**A**), BSG (**B**), DPP4 (**C**), AGTR2 (**D**), ANPEP (**E**), cathepsin B (**F**), and cathepsin L (**G**) were measured by q-PCR. GAPDH was used as a housekeeping. Graphs indicate the mean ± SD from three independent experiments. ****p < 0.0001, ***p = 0.0002, **p = 0.003, *p < 0.0449, *n* = 3–6 per group

The highest expression of ADAM17 mRNA was found in SH-SY5Y cells, followed by astrocytes, microglial cells, and EC and pericytes, which expressed the same levels of ADAM17 mRNA (Fig. 3A). The highest BSG mRNA levels were also detected in SH-SY5Y cells, followed by microglial cells, pericytes, astrocytes, and EC (Fig. 3B). DPP4 mRNA levels were the highest in microglial cells, followed by SH-SY5Y5 cells, EC and pericytes with the same DPP4 mRNA expression, and astrocytes that exhibited the lowest expression (Fig. 3C). SH-SY5YSY-Y5 and microglial cells expressed the most pronounced AGTR2 mRNA levels, followed by pericytes, astrocytes, and EC (Fig. 3D). In the case of ANPEP, the highest mRNA expression was observed in SH-SY5YSY-Y5 cells, followed by pericytes, EC, microglial cells, and astrocytes (Fig. 3E). Cathepsin B mRNA presented the most prominent levels in astrocytes and SH-SY5YSY-Y5 cells, followed by EC, pericytes, and microglial cells, which expressed this gene at the same level (Fig. 3F). Lastly, the highest expression of cathepsin L was found in SH-SY5YSY-Y5 cells, followed by pericytes, microglial cells, astrocytes, and EC (Fig. 3G).

### Claudin-5 and ZO-1 expression levels are influenced by exposure of the S1 subunit in primary human brain EC

EC are essential cells forming the BBB [63]. Given our previous results showing that ACE2 protein and mRNA levels are lower in EC and pericytes when compared to other NVU cells, we investigated if EC can be affected by exposure to the S1 subunit of the SARS-CoV-2 S protein, the domain responsible for direct binding to the ACE2 receptor [19]. We analyzed TJ protein expression changes, as their alterations may provide transendothelial entry of SARS-CoV-2 into the brain.

**Fig. 4:**
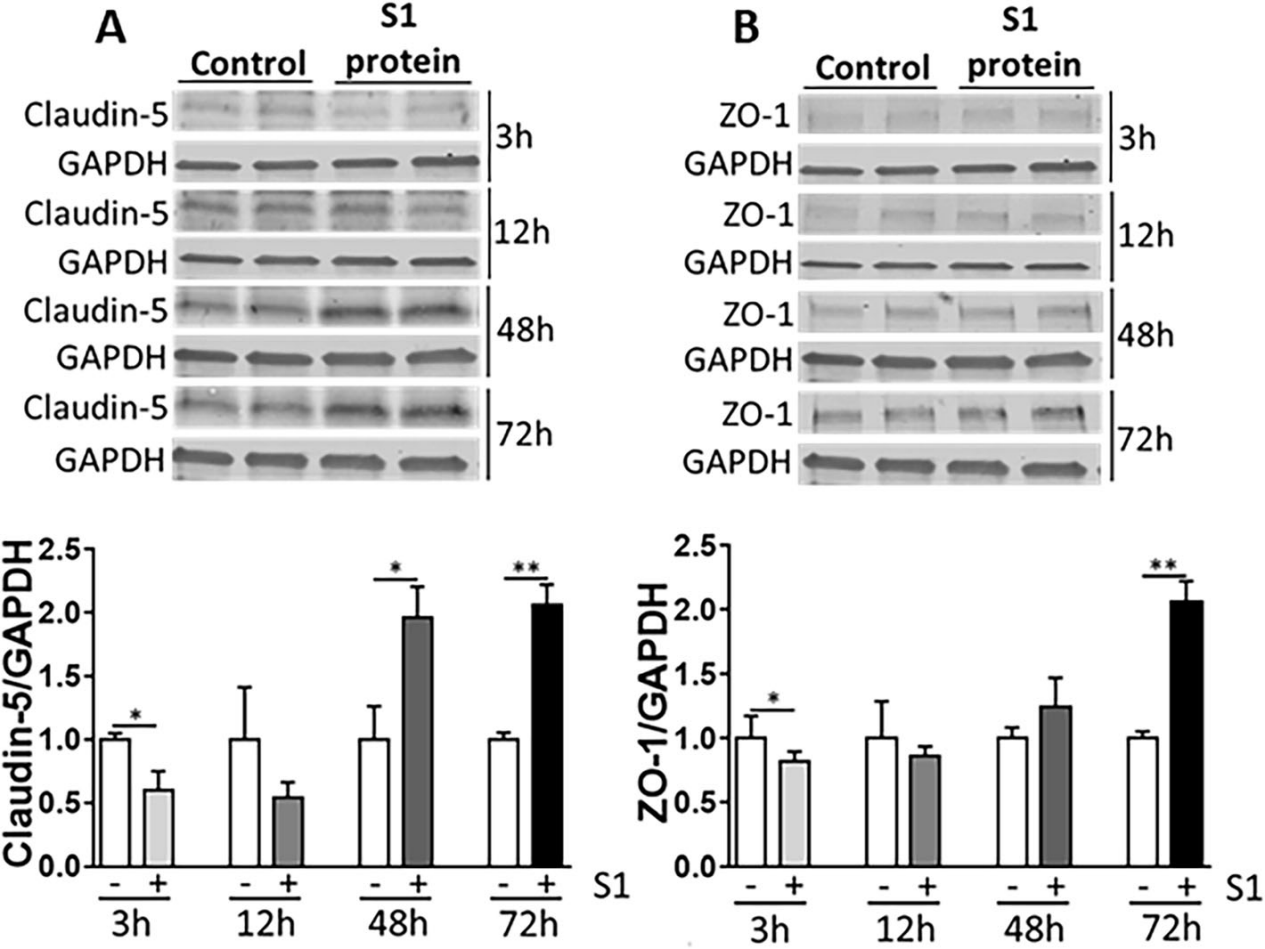
Impact of the SARS-CoV-2 S1 protein subunit on claudin-5 and ZO-1 expression in EC. Human primary endothelial cells were exposed to 15 nM of the SARS-CoV-2 S1 protein subunit for 3, 12, 48, or 72 h and the expression levels of claudin-5 (**A**) and ZO-1 (**B**) were measured by immunoblotting. GAPDH was used as a loading control. Graphs indicate the mean ± SD. *p < 0.0449, **p = 0.003; *n* = 3 per group, three independent experiments

Human EC were exposed to a single dose of 15 nM of the SARS-CoV-2 S1 subunit and the expression levels of claudin-5 and ZO-1, two key TJ proteins, were analyzed by immunoblotting. The claudin-5 expression level was significantly lower when cells were exposed to the S1 subunit for 3 h as compared with untreated cells. Interestingly, there was a significant upregulation in the levels of claudin-5 when compared with the controls after 48 h and 72 h, suggesting recovery processes (Fig. 4A). We also observed a downregulation in ZO-1 protein after 3 h of exposure, followed by a significant increase after 72 h of exposure as compared with the untreated cells (Fig. 4B).

### ACE2 and TMPRSS2 expression levels increase after HIV-1 infection in primary human brain astrocytes

**Fig. 5:**
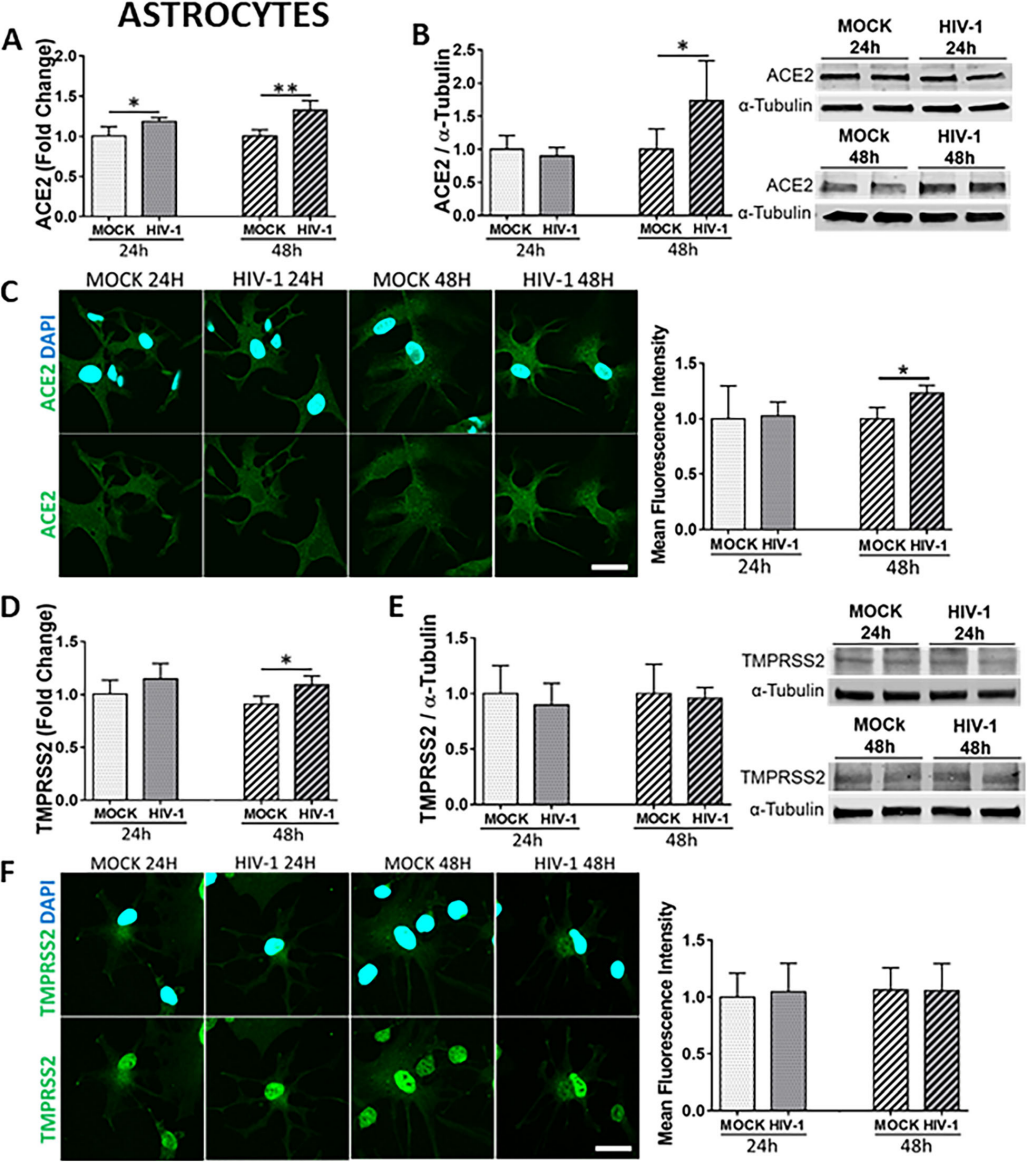
Impact of HIV-1 infection on ACE2 and TMPRSS2 expression in astrocytes. Human primary astrocytes were either mock-infected or infected with HIV-1 with 60 ng/mL HIV-1 p24 for 24 h or 48 h and the expression levels of ACE2 and TMPRSS2 were measured by q-PCR (**A** and **D**, respectively), immunoblotting (**B** and **E**, respectively), and immunostaining (**C** and **F**, respectively). GAPDH was used as a housekeeping gene and α-tubulin as a loading control. Graphs indicate the mean ± SD from three independent experiments. **p = 0.003, *p < 0.0449, *n* = 4–5 per group; scale bars, 30 μm

Astrocytes are the most abundant cell type of the CNS [64]. Although it has been reported that astrocytes do not express the classical CD4 receptor for HIV-1 entry [65], several groups have identified HIV infection in astrocytes in vivo [51, 53–55, 57, 66]. While some early studies described a non-productive infection [67], other reports indicated productive HIV-1 replication in astrocytes at low levels [51, 68] by the mechanisms that involve exosomes from infected trafficking CD4 cells or from direct cell to cell contact [69]. We also detected HIV-1 gag RNA expression and p24 levels in astrocyte cultures as the result of HIV-1 infection (Supplemental Fig. 2 A and B, respectively). The expression of ACE2 and TMPRSS2 was measured by qPCR, immunoblotting, and immunostaining in mock-infected cultures and cultures infected with 60 ng/ml of HIV-1 for 24 h and 48 h. ACE2 mRNA and protein levels increased in astrocytes as the result of HIV-1 infection. ACE2 mRNA levels were elevated at both 24 h and 48 h post infection (Fig. 5A), while ACE2 protein levels increased only 48 h post infection (Fig. 5 B and C). In addition, TMPRSS2 mRNA levels were increased at 48 h post HIV infection (Fig. 5D); however, no changes were found at the protein level (Fig. 5 E and F).

### No changes in ACE2 or TMPRSS2 expression are detected after HIV-1 infection in primary human brain pericytes

**Fig. 6:**
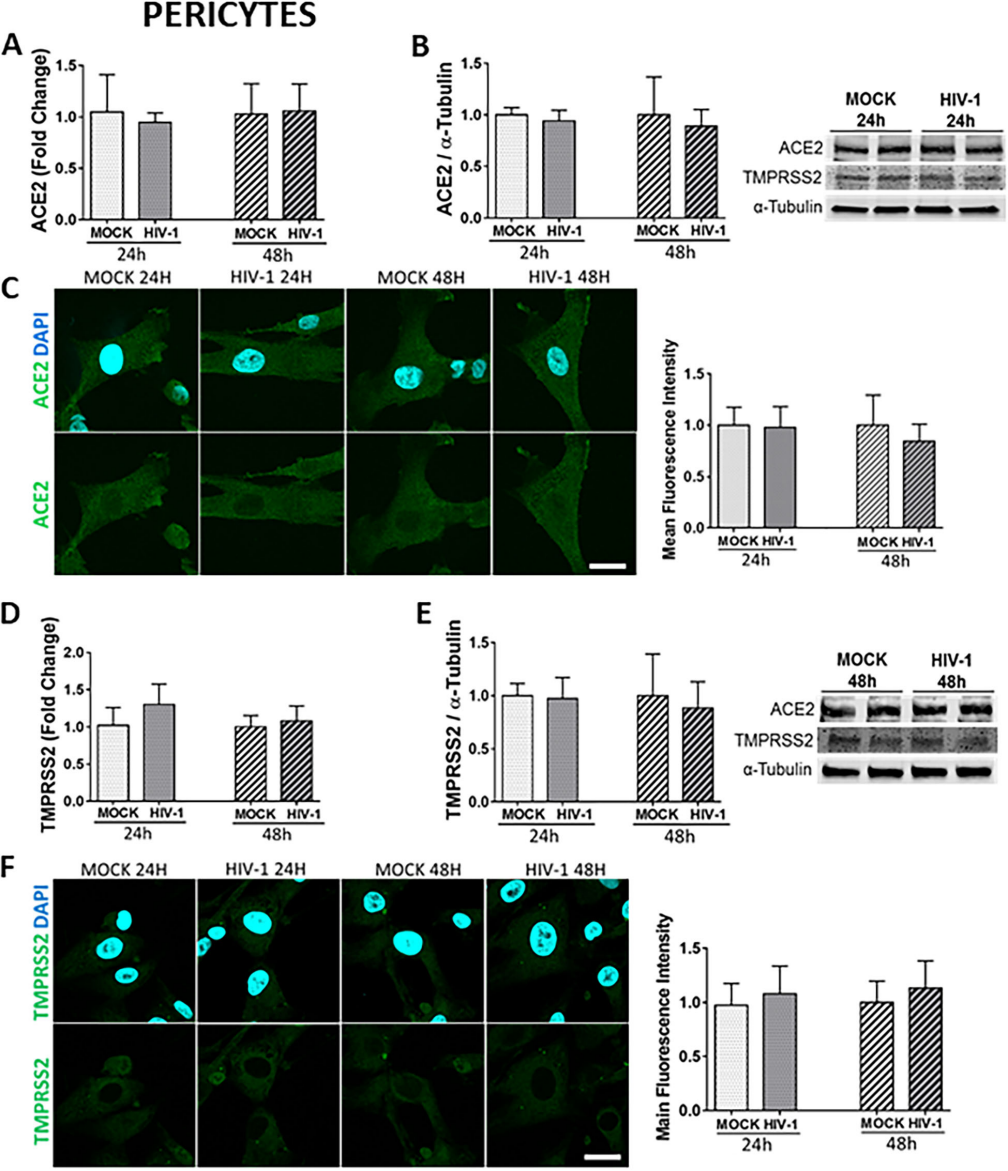
Impact of HIV-1 infection on ACE2 and TMPRSS2 expression in pericytes. Human primary pericytes were either mock-infected or infected with HIV-1 as in Fig. 5 and the expression levels of ACE2 and TMPRSS2 were measured by q-PCR (**A** and **D**, respectively), immunoblotting **(B** and **E**, respectively), and immunostaining (**C** and **F**, respectively). GAPDH was used as a housekeeping gene and α-tubulin as a loading control. Graphs indicate the mean ± SD from three independent experiments; *n* = 4 per group; scale bars, 30 μm

Brain pericytes express the main HIV-1 receptors, CD4, CCR5, and CXCR4, and can be efficiently infected by HIV-1 [54, 58], which was confirmed in the present study by the assessment of HIV-1 gag RNA and p24 levels (Supplemental Figures 2C and 2D, respectively). We then investigated if HIV-1 infection could lead to alterations of ACE2 or TMPRSS2 expression in pericytes. Infection with 60 ng/ml of HIV-1 for 24 h or 48 h did not result in any changes in the expression of ACE2 or TMPRSS2 at the mRNA (Fig. 6 A and D, respectively) or protein levels (Fig. 6 B, C, E, and F) in these cells.

### HIV-1 infection of human microglial cells upregulates ACE2 and TMPRSS2 expression

**Fig. 7:**
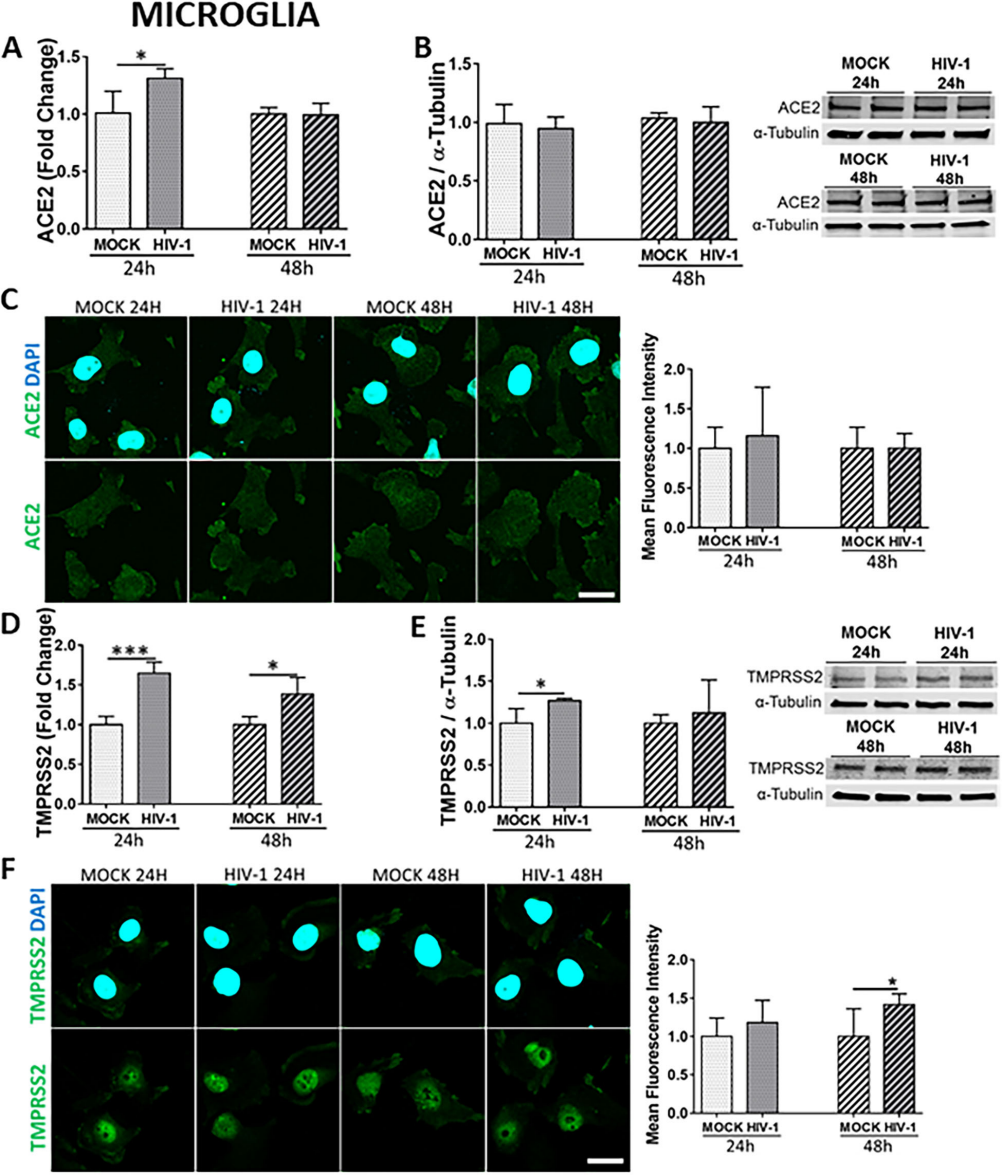
Impact of HIV-1 infection on ACE2 and TMPRSS2 expression in microglial cells. Microglia were either mock-infected or infected with HIV-1 as in Fig. 5 and the expression levels of ACE2 and TMPRSS2 were measured by q-PCR (**A** and **D**, respectively), immunoblotting (**B** and **E**, respectively), and immunostaining (**C** and **F**, respectively). GAPDH was used as a housekeeping gene and α-tubulin as a loading control. Graphs indicate the mean ± SD from three independent experiments. ***p = 0.0002, *p < 0.0449, *n* = 4 per group; scale bars, 30 μm

Microglia are the main cell type supporting productive HIV-1 infection and are regarded as one of the major cellular reservoirs of latent HIV-1 in the brain [49, 70–73]. We also detected HIV-1 gag RNA expression and p24 levels in these cells after HIV-1 infection (Supplemental Figures 2E and 2F). HIV-1 infection resulted in a significant increase in the ACE2 mRNA level at 24 h but not at 48 h as compared to mock-infected cells (Fig. 7A). However, no changes in ACE2 expression were detected at the protein level (Fig. 7 B and C). The expression pattern of TMPRSS2 indicated a significant increase at the mRNA levels at both 24 h and 48 h post infection compared to mock-infected cells (Fig. 7D). In addition, a significant increase in the TMPRSS2 protein level was observed post HIV-1 infection (Fig. 7 E and F).

## Discussion

Neurological complications are frequent in patients affected by COVID-19 [4, 5]. Several studies have reported that SARS-CoV-2 can invade the CNS; however, the mechanisms of this process remain unclear. It was proposed that an invasion of the CNS by infection of the BBB cells may be responsible for this effect [11, 39, 74]. SARS-CoV-2 entry into the cells uses the ACE2 receptor and the serine protease TMPRSS2 to allow the interaction with the viral spike protein S, followed by a membrane fusion resulting in cell viral entry [20]. In addition, a variety of other molecules have been suggested to be involved in the SARS-CoV-2 internalization into the host cell. Some of them are ADAM17 [23], DPP4 [25], AGTR2 [27], BSG [19], ANPEP [30], and cathepsin B/L [31].

Specific cells of the NVU that can be implicated in SARS-CoV-2 neuroinvasion have not been identified. In particular, a comprehensive profile of the host receptors on the NVU cells that can be involved in SARS-CoV-2 entry into the brain is unknown. Such studies are important, because they can identify which of the NVU cell types may provide the main route of SARS-CoV-2 entry into the CNS. Previous studies indicated that astrocytes, microglial cells, and endothelial cells express ACE2 [29, 75, 76]. Recently, high expression levels of ACE2 in adult human heart pericytes [77, 78] and mouse olfactory bulb pericytes [79] have been reported. However, brain pericytes have a different origin and no studies have yet described the expression of ACE2 in human BBB pericytes. Of the NVU cells, expression of TMPRSS2 has only been studied and reported in microglial cells [29]. Studies have shown a low level of expression of TMPRSS2 in blood vessel endothelial cells; however, there are no reports of TMPRSS2 expression in microvascular endothelial cells that compose the BBB [80].

The current study describes the profile of expression of the main receptors involved in SARS-CoV-2 infection and entry into the NVU cells. Our results indicated that SH-SY5YSY-Y5 neuroblastoma cells displayed the highest mRNA expression of ACE2 and TMPRSS2; however, they presented the lowest protein expression. SH-SY5Y also shows the highest mRNA expression levels for ADAM17, BSG, ANPEP, and cathepsin L. These results may suggest that neurons might be susceptible to SARS-CoV-2 infection. Nevertheless, the evidence implicates that SARS-CoV-2 infects mostly vascular and immune cells in the human brain rather than directly infecting neurons [81–83].

Astrocytes exhibited the highest level of cathepsin B as well as the second highest levels of ACE2, TMPRSS2, and ADAM17. They also expressed mRNA for BSG, AGTR2, and cathepsin L but the lowest mRNA expression for DPP4 and ANPEP. Among the cells of the NVU, prominent mRNA levels for ACE2 were detected in microglial cells; however, their ACE2 protein level expression was relatively low. Microglial cells also exhibited the highest levels of DPP4 and AGTR2 mRNA. mRNA levels for TMPRSS2, BSG, ADAM17, ANPEP, and cathepsin B and L were also prominently expressed in these cells. These results support literature reports showing ACE2 and TMPRSS2 mRNA and protein levels in human brain microglial cells [29]. Microglia were also reported to express ADAM17 [84] and cathepsin B and L [85]. Differential expression of the major SARS-CoV-2 cell entry molecules is likely to make cells of the NVU, especially astrocytes and microglial cells, uniquely susceptible to SARS-CoV-2 infection.

EC and pericytes expressed ACE2 and TMPRSS2 mRNA at low levels as compared to astrocytes, SH-SY5YSY-Y5, and microglial cells. Similar to TMPRSS2 mRNA, BSG, AGTR2, and cathepsin L mRNA levels in EC were the lowest among studied cells of the NVU. These findings are important because EC generate the main interface between the bloodstream and the brain. Thus, a low expression of ACE2 and TMPRSS2 may provide some protection against SARS-CoV-2 entry into the brain. On the other hand, protein expression of ACE2 and TMPRSS2 in EC was higher and comparable to other cells of the NVU. We next analyzed if ACE2 expression on EC can induce phenotype changes upon exposure to the S1 subunit of the SARS CoV-2 S protein, the domain responsible for direct binding to the ACE2 receptor [19]. EC treatment with the S1 subunit resulted in TJ dual-stage response pattern, where claudin-5 and ZO-1 expression levels decreased 3 h after exposure, followed by an increase after 48 h and 72 h as compared with the controls (Fig. 4). Disruption of TJ protein expression in EC exposed to the S1 subunit is consistent with observations that the S protein alters barrier function in a model of the human blood-brain barrier [86].

Within the NVU, EC closely interact with pericytes. Indeed, pericytes wrap around the brain endothelium via cytoplasmic processes that extend along the abluminal surface of the endothelium and cover close to 100% of the brain microvascular endothelium. Part of the pericyte-endothelial interface is separated by the basement membrane; however, pericytes also remain in direct contact with endothelial cells via the peg-socket type of arrangement [56, 87]. Therefore, it was important that mRNA expression of ACE2 and TMPRSS2 was also low in pericytes.

Several studies have focused on investigating a possible association between ACE2 expression and the interferon (IFN)-signaling pathway. It was reported that the administration of exogenous IFN-γ downregulated the expression of ACE2 receptors in interferon-deficient Vero E6 cells [88]. Interestingly, recent reports suggested ACE2 to be one of the interferon-stimulated genes (ISG) [20, 89, 90]. Indeed, it was shown that type I IFNs, and to a lesser extent type II IFNs, can significantly upregulate ACE2 expression levels in human nasal epithelial cells. In addition, type I IFNs can upregulate ACE2 in other cells of the epithelial barrier tissue, such as primary bronchial cells and keratinocytes [89]. The finding that ACE2 is an ISG has broad implications, including HIV infection. In fact, HIV-1 entry into host cells stimulates an IFN-driven induction of ISGs as part of the cellular antiviral defense network [91].

The CNS is susceptible to infection by lentiviruses, such as HIV-1, through the viral entry from the periphery into the brain [74, 92]. Several studies have shown that HIV-1 infection modulates gene and protein expression in the host cells [54, 91]. Therefore, we proposed to evaluate whether HIV-1 infection modulates the expression levels of ACE2 and TMPRSS2. We first examined the efficiency of HIV-1 infection of NVU cells. Primary human brain astrocytes, pericytes, and human microglial cells were infected with HIV-1 for 24 h and 48 h. EC and SH-SY5YHSY-Y5 neuroblastoma cells were excluded from these analyses because no productive HIV-1 infection in EC or neurons has been reported [59, 60]. In agreement with previous studies, we confirmed a successful infection of HIV-1 in microglial cells [49, 50], pericytes [54–56], and astrocytes [51–53] by the measurement of HIV-1 gag and p24 levels qPCR and ELISA, respectively. The expression of HIV-1 gag 24 h after infection was significantly higher compared to 48 h in microglia, in pericytes, and, to a lesser degree, in astrocytes, suggesting early induction of anti-HIV mechanisms (Supplemental Figure 2).

Next, we evaluated if ACE2 and/or TMPRSS2 levels are affected by HIV-1 infection. Overall, a significant increase in ACE2 and TMPRSS2 at both mRNA and protein levels was observed in HIV-1 infected astrocytes and, especially, in microglial cells (Figs. 5 and 7). These effects may be related to IFN-α/β signaling that was reported to regulate HIV infection in both microglia and astrocytes [93, 94]. Indeed, astrocytes and microglia are the main producers of IFN during inflammatory response in the CNS [95]. Microglia are also the cell type that is most susceptible to HIV-1 infection within the CNS.

In contrast, no changes in ACE2 or TMPRSS2 mRNA or proteins were detected in pericytes upon HIV infection (Fig. 6), even though pericytes can be productively infected by HIV-1 and respond to inflammatory signals [96–98]. On the other hand, HIV-1 infection in pericytes appears to not be influenced by IFNs as interferon-α, interferon-β, and interferon-γ levels were not affected in HIV-infected pericytes [55]. These results may confirm the notion that ACE2 expression is regulated by IFNs upon HIV-1 infection. In support of this notion, an increase in ACE2 expression in secretory cells of the nasal epithelium has been reported in infection by the influenza virus [89]. The influenza virus is recognized to be an efficient inducer of the IFN pathway similar to HIV-1 [99]. An overexpression of ACE2 mRNA in CD4+T cells has also been described in patients with systemic lupus erythematosus [100], a disease that is associated with interferon induction [101, 102].

Elevated COVID-19 mortality in patients with immunocompromised immune systems [103] suggests that people with HIV-1 might be at an increased risk of COVID-19-related complications and death. Surprisingly, several studies indicated that COVID-19 pathology does not markedly differ between HIV-1-infected individuals and the general population [104–108]. These findings can be explained as the result of successful antiretroviral therapy (ART) that decreases plasma HIV-1 viral load to undetectable levels [109–112]. On the other hand, ART is less efficient in the treatment of HIV-1 infection in the brain due to the barrier function of the BBB, which limits brain penetration of antiretroviral drugs. Thus, the interactions between HIV-1 and COVID-19 in the CNS remain a threat. In addition, HIV-1-infected patients who are not on ART might be at increased risk of SARS-CoV-2 infection and more severe COVID-19 outcomes.

In conclusion, the present study describes the coexpression of the main receptors involved in SARS-CoV-2 infection in the cells of the NVU, suggesting their susceptibility to SARS-CoV-2 infection. Among NVU cells, the most prominent expression of SARS-CoV-2 receptors was observed in astrocytes and microglial cells. Additionally, HIV-1 infection of brain astrocytes and microglia cells upregulated ACE2 and TMPRSS2 expression levels. These findings will help to better understand the pathology of CNS infection by SARS-CoV-2 and the role of HIV-1 infection in the progression of COVID-19.

## Supplementary Information

**Additional file 1 **. Supplemental Figure 1. HIV-1 infection does not affect viability of the NVU cells. Cell viability was examined by the trypan blue exclusion assay (**A, C, E**) and the CCK-8 assay (**B, D, F**). Graphs indicate the mean ± SD from three independent experiments. n= 4-6 per group. Supplemental Figure 2. HIV-1 gag expression by the NVU cells and HIV-1 p24 secretion into cell culture media as the result of HIV-1 infection. Astrocytes, pericytes, and microglial cells were either mock-infected or infected with 60 ng/mL HIV-1 p24 for 24h or 48h. The expression of HIV-1 gag was measured by qPCR (A, C, E) and HIV-p24 by ELISA (B, D, F). Graphs indicate the mean ± SD from three independent experiments. ****p<0.0001, ***p=0.0002, **p=0.003. *n*= 3-6 per group.

## Data Availability

All data generated during and/or analyzed during the current study are included in this published article. All source data supporting the findings of this manuscript are available from the corresponding authors upon request.

## Abbreviations

ACE2: Angiotensin I converting enzyme 2
ADAM17: A disintegrin and metalloproteinase 17
AGTR2: Angiotensin II receptor type 2
ANPEP: Aminopeptidase N
BBB: Blood-brain barrier
BCA: Bicinchoninic acid
BSG: Basigin
CNS: Central nervous system
COVID-19: Coronavirus disease-19
DPP4: Dipeptidyl peptidase 4
EC: Endothelial cells
FBS: Fetal bovine serum
GAPDH: Glyceraldehyde 3-phosphate dehydrogenase
HEK: Human embryonic kidney
HIV-1: Human immunodeficiency virus
IFN: Interferon
ISG: Interferon-stimulated genes
NVU: Neurovascular units
PBS: Phosphate-buffered saline
qPCR: Quantitative PCR
S protein: Spike protein
S1: S1 Subunit of the spike protein
SARS-CoV-2: Severe acute respiratory syndrome-coronavirus 2
TMPRSS2: Transmembrane protease, serine 2
ZO-1: Zonula occludens-1
TJ: Tight junction
