# Supplementary material for: Expression of SARS-CoV-2-related receptors in cells of the neurovascular unit: implications for HIV-1 infection
Source: J Neuroinflammation. 2021 Jul 29;18:167. doi: 10.1186/s12974-021-02210-2 (PMC8319595; doi:10.1186/s12974-021-02210-2)
Supplement: Supplementary file 1 — Additional file 1 . Supplemental Figure 1. HIV-1 infection does not affect viability of the NVU cells. Cell viability was examined by the trypan blue exclusion assay (A, C, E) and the CCK-8 assay (B, D, F). Graphs indicate the mean ± SD from three independent experiments. n= 4-6 per group. Supplemental Figure 2. HIV-1 gag expression by the NVU cells and HIV-1 p24 secretion into cell culture media as the result of HIV-1 infection. Astrocytes, pericytes, and microglial cells were either mock-infected or infected with 60 ng/mL HIV-1 p24 for 24h or 48h. The expression of HIV-1 gag was measured by qPCR (A, C, E) and HIV-p24 by ELISA (B, D, F). Graphs indicate the mean ± SD from three independent experiments. ****p<0.0001, ***p=0.0002, **p=0.003. n= 3-6 per group. [file 12974_2021_2210_MOESM1_ESM.pdf]

Expression of SARS-CoV-2-related Receptors in Cells of the Neurovascular Unit: Implications for HIV-1 infection

Silvia Torices[1], Rosalba Cabrera[1], Michael Stangis[1], Oandy Naranjo[1], Nikolai Fattakhov[1], Timea Teglas[1], Daniel Adesse[2], Michal Toborek[1]

[1]Department of Biochemistry and Molecular Biology, University of Miami Miller School of Medicine, Miami, FL, 33136, USA. [2]Laboratory of Structural Biology, Instituto Oswaldo Cruz, Fiocruz, Rio de Janeiro, RJ, CEP 21045-900, Brazil

SUPPLEMENTAL DATA

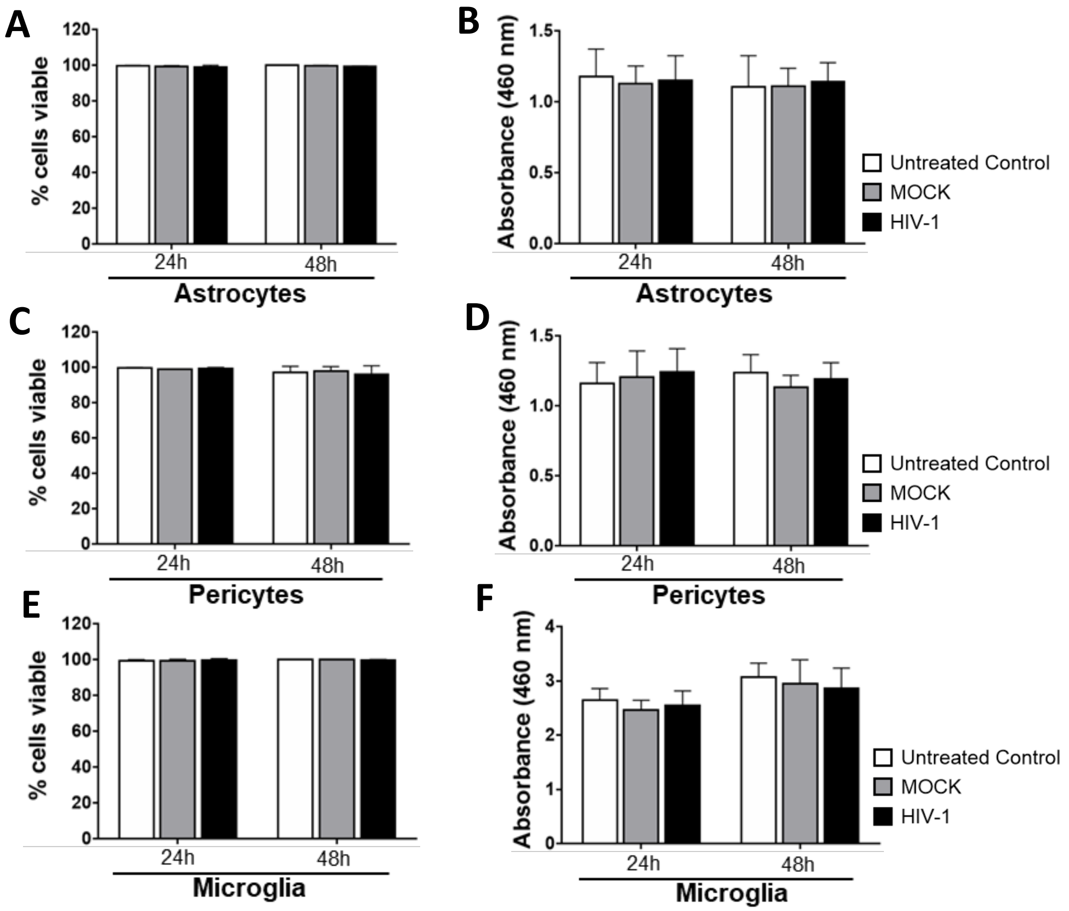

Supplemental Figure 1. HIV-1 infection does not affect viability of the NVU cells. Cell viability was examined by the trypan blue exclusion assay (**A, C, E**) and the CCK-8 assay (**B, D, F**). Graphs indicate the mean ± SD from three independent experiments. n= 4-6 per group.

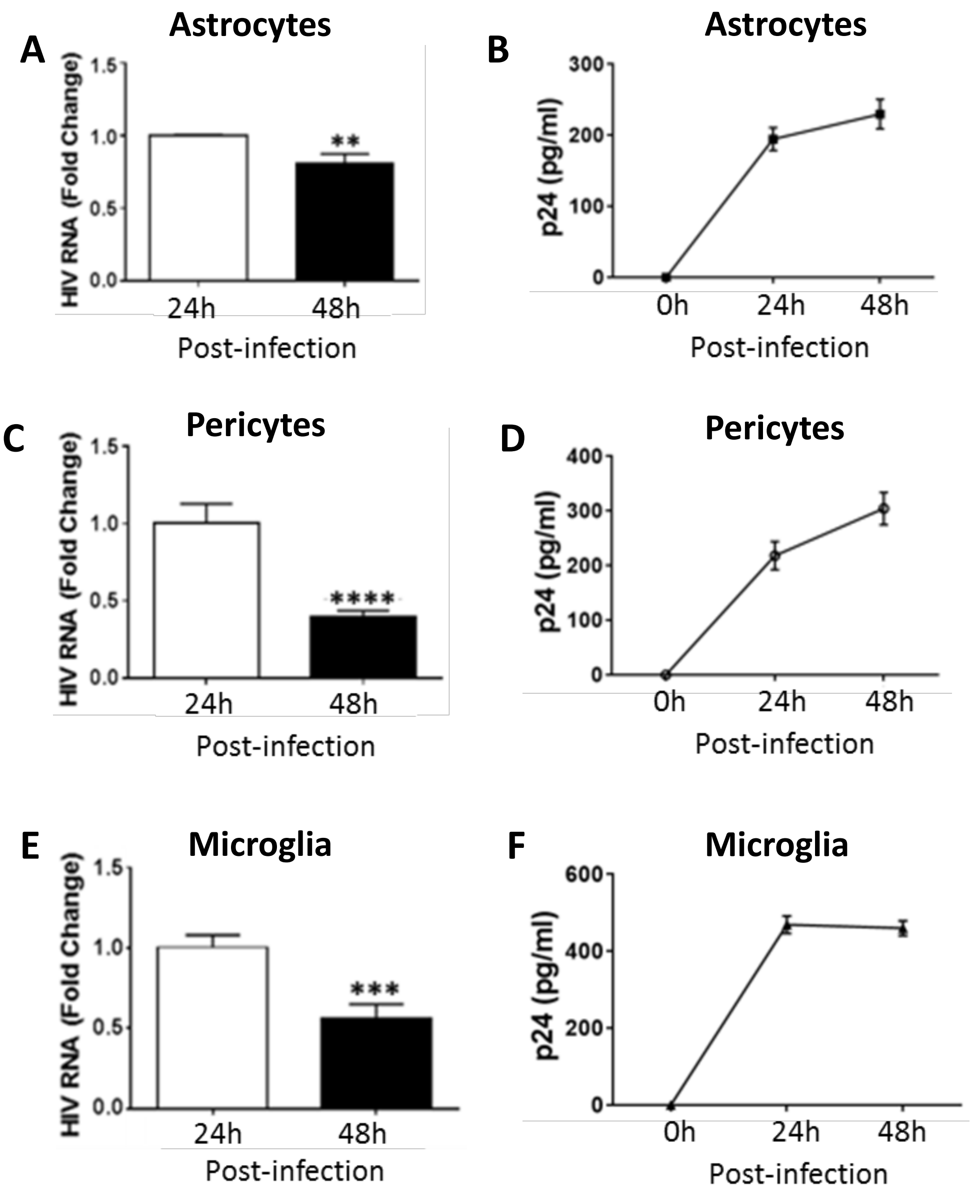

Supplemental Figure 2. HIV-1 gag expression by the NVU cells and HIV-1 p24 secretion into cell culture media as the result of HIV-1 infection. Astrocytes, pericytes, and microglial cells were either mock-infected or infected with 60 ng/mL HIV-1 p24 for 24h or 48h. The expression of HIV-1 gag was measured by qPCR (A, C, E) and HIV-p24 by ELISA (B, D, F). Graphs indicate the mean ± SD from three independent experiments. ****p<0.0001, ***p=0.0002, **p=0.003. n= 3-6 per group.
